# Machine learning immune-related gene based on KLRB1 model for predicting the prognosis and immune cell infiltration of breast cancer

**DOI:** 10.3389/fendo.2023.1185799

**Published:** 2023-06-07

**Authors:** Guo Huang, Shuhui Xiao, Zhan Jiang, Xue Zhou, Li Chen, Lin Long, Sheng Zhang, Ke Xu, Juan Chen, Bin Jiang

**Affiliations:** ^1^ Hengyang Medical School, University of South China, Hengyang, Hunan, China; ^2^ The Second Affiliated Hospital, Department of Breast and Thyroid Surgery, Hengyang Medical School, University of South China, Hengyang, Hunan, China; ^3^ Department of Oncology, Chongqing General Hospital, Chongqing, China; ^4^ Department of Oncology, The Affiliated Hospital of Southwest Medical University, Luzhou, China; ^5^ Department of Ultrasonography, Chengdu First People's Hospital, Chengdu, China; ^6^ Department of Radiology, Nanchong Central Hospital, The Second Clinical Medical College, North Sichuan Medical College, Nanchong, China; ^7^ The Second Affiliated Hospital, Department of Radiotherapy, Hengyang Medical School, University of South China, Hengyang, Hunan, China; ^8^ The Second Affiliated Hospital, Department of Burn and Plastic Surgery, Hengyang Medical School, University of South China, Hengyang, Hunan, China

**Keywords:** breast cancer, KLRB1, prognostic model, immune-related gene, immune infiltration

## Abstract

**Objective:**

Breast cancer is a prevalent malignancy that predominantly affects women. The development and progression of this disease are strongly influenced by the tumor microenvironment and immune infiltration. Therefore, investigating immune-related genes associated with breast cancer prognosis is a crucial approach to enhance the diagnosis and treatment of breast cancer.

**Methods:**

We analyzed data from the TCGA database to determine the proportion of invasive immune cells, immune components, and matrix components in breast cancer patients. Using this data, we constructed a risk prediction model to predict breast cancer prognosis and evaluated the correlation between KLRB1 expression and clinicopathological features and immune invasion. Additionally, we investigated the role of KLRB1 in breast cancer using various experimental techniques including real-time quantitative PCR, MTT assays, Transwell assays, Wound healing assays, EdU assays, and flow cytometry.

**Results:**

The functional enrichment analysis of immune and stromal components in breast cancer revealed that T cell activation, differentiation, and regulation, as well as lymphocyte differentiation and regulation, play critical roles in determining the status of the tumor microenvironment. These DEGs are therefore considered key factors affecting TME status. Additionally, immune-related gene risk models were constructed and found to be effective predictors of breast cancer prognosis. Further analysis through KM survival analysis and univariate and multivariate Cox regression analysis demonstrated that KLRB1 is an independent prognostic factor for breast cancer. KLRB1 is closely associated with immunoinfiltrating cells. Finally, *in vitro* experiments confirmed that overexpression of KLRB1 inhibits breast cancer cell proliferation, migration, invasion, and DNA replication ability. KLRB1 was also found to inhibit the proliferation of breast cancer cells by blocking cell division in the G1/M phase.

**Conclusion:**

KLRB1 may be a potential prognostic marker and therapeutic target associated with the microenzymic environment of breast cancer tumors, providing a new direction for breast cancer treatment.

## 1 Introduction

Breast cancer (BC) is the most predominant type of carcinoma in women, comprising 30% of all cancers affecting females and a mortality rate of around 15% (1). However, primary breast tumors alone are not the leading cause of death in BC patients, while drug resistance, recurrence, and metastasis are the leading causes of increased mortality. Following the onset of metastasis, the survival rate after five years is merely 25% (2). Around 2.09 million new cases of BC were reported globally in 2018, of which roughly 620,000 individuals died due to the disease. The incidence of BC is highest among Chinese women. The projected instances of BC in China are expected to rise to 2.5 million by 2021 (3).

The colonization of tumor cells in normal tissues, the stromal cells, and immune cells that coexist with these tumor cells and the factors they secrete, the vascular endothelial cells, and the extracellular matrix collectively form the tumor microenvironment (TME) (4). The tumor cells prompt the recruitment and activation of immune cells and stromal components, creating a tumor-suppressive inflammatory microenvironment during the initial tumor colonization or growth stages. As a result, this microenvironment impedes tumorigenesis and the advancement of the tumor. However, following sustained stimulation by tumor antigens and immune activation responses, the pertinent effector cells within the TME become exhausted or remodeled, rendering them incapable of fulfilling their usual functions or even facilitating the malignant manifestations of tumors. This, in turn, results in the formation of an immunosuppressive microenvironment.

These components are crucial in tumorigenesis, development, and immune escape (5). Tumor-associated macrophages can be polarized in TME as M1 type (classical activation) or M2 type (alternative activation). M1-type macrophages have a pro-inflammatory phenotype and inhibit tumor growth and metastasis by secreting associated inflammatory cytokines such as tumor necrosis factor-α (TNF-α) and interleukin-1β (IL-1β), which can be induced *in vitro* by LPS or IFN-γ (6, 7). M2-type macrophages, on the other hand, are immunosuppressive phenotypes that induce tumor cell invasion and migration by secreting immunosuppressive factors such as IL-10, which can be induced *in vitro* by IL-4 (8). Moreover, research has verified that the extent of immune cell infiltration is associated with the prognosis of individuals with cancer. Hence, evaluating the heterogeneity of TME and remodeling the immune microenvironment of tumors may represent a new avenue for treating cancer (9).

Killer cell lectin-like receptor B1 (KLRB1), encodes CD161, which belongs to the C-type lectin family and was initially defined as a receptor for natural killer (NK) cells. It was later discovered to also exist in subpopulations of CD4+ and CD8+ T cells (10). The attachment of CD161 on T cells provides a co-stimulatory signal for T cell receptor (TCR)-mediated activation (11). KLRB1 transcription is repressed in approximately 68% of people with non-small cell lung carcinoma, suggesting that KLRB1 could serve as a predictive tumor marker (12). In this study, identifying differentially expressed genes (DEGs) for stromal and immune components in cases of BC revealed that KLRB1 could be a potential marker affecting the tumor microenvironment of BC.

## 2 Materials and methods

### 2.1 Collection and preprocessing of BC data

The TCGA (https://portal.gdc.cancer.gov/repository) database was utilized to acquire BC mRNA data and their corresponding clinical data from 1109 patients, which were then presented in a standardized FPKM format.

### 2.2 ESTIMATE

The “Estimate” R package was utilized to calculate Stromal, Immune, and ESTIMATE scores (13).

### 2.3 DEGs identification according to stromal and immune scores

The 1109 BC cases were categorized into high- and low-score subgroups per the median comparison of stromal and immune scores and the DEGs between the two were determined through the Limma R package. The |log_2_FC|>1 and *p*<0.05 served as the identification criteria. Heat maps were drawn using the R package”pheatmap” (14).

### 2.4 Functional enrichment analysis

The DEGs co-expressed genes of Stromal score and Immune score were obtained using Venn diagrams for GO and KEGG (15) enrichment analysis.

### 2.5 Identification of potential prognostic DEGs with univariate Cox models

The LASSO Cox regression narrowed the range of prognostic DEGs to reduce the risk of overfitting (16). Multivariate Cox regression selected the DEGs most closely associated with survival which were utilized to construct risk models to predict patient survival. By using the standardized expression levels of all genes and their regression coefficient, the risk score for each patient was computed.


$$\begin{array}{c} \text{Risk score}=\text{FOLR}2\times 0.016+\text{PEX}5\text{L}\times 0.077+\text{KLRB}1\times -0.171\\ +\text{EPYC}\times 0.041+\text{BHLHE}22\times 0.064 \end{array}$$

The data were visualized in two dimensions utilizing principal component analysis (PCA) and t-distribution random neighborhood embedding (t-SNE) analysis through the “Rtsne” and “ggplot2” software packages. Furthermore, univariate and multivariate Cox regression analyses were conducted, and independent prognostic factors were identified using the “survival” package.

### 2.6 Building a prediction atlas

By selecting five independent predictive genes, a prediction atlas was constructed, and the robustness of the prediction model was assessed at 1-, 2-, and 3- years (17). The prediction atlas was corrected using calibration charts through a guided method of 1000 resamplings.

### 2.7 Gene set enrichment analysis

The R package “gsva” was utilized for single sample gene set concentration analysis (ssGSEA) to determine the signaling pathways that may be linked to both KLRB1 expression groups (18).

### 2.8 Estimation of TICs

The proportion of 22 TICs in BC samples was calculated utilizing the CIBERSORT algorithm (19), and the results were presented as bar graphs. The proportion of immune cells in tumor tissues with enhanced and reduced expression of KLRB1 was compared utilizing the Wilcoxon rank sum test, and the correlation between the proportion and KLRB1 expression was assessed.

### 2.9 KLRB1 differential expression and survival analysis

To compare the expression of KLRB1 mRNA between BC tissues and normal tissues, the Wilcoxon rank sum test was utilized, and the outcomes were visualized with the “ggpubr” R package. Survival of high and low KLRB1 expression was analyzed using the “survival” R package.

### 2.10 Drug sensitivity analysis and immunotherapy

In order to observe the differences in efficacy of chemotherapy drugs based on KLRB1 expression, we utilized the “pRophetic” package to calculate the half-maximal inhibitory concentration (IC50) of commonly used drugs for treating breast cancer (20). Moreover, we conducted an analysis of the correlation between KLRB1 expression and immunotherapy for breast cancer, utilizing the TCIA database.

### 2.11 Cell culture

Breast normal epithelial cell (MCF-10A) and breast cancer cell lines (MCF7, Hs 578T, HCC1937, MDA-MB-231) were retrieved from ATCC (Manassas, USA). MCF-10A, MCF7, and MDA-MB-231 cells were grown in DMEM high sugar medium (Gibico, China). HCC1937 cells were grown in 1640 medium (Gibico, China). The medium was supplemented with 10% FBS (Pricells, China) and 1% penicillin-streptomycin solution (Solarbio, China). Afterward, the cells were put in an incubator containing 5% CO2 at 37°C.

### 2.12 Cell infection with lentivirus

Once the cells attached and reached a density of 30%, MCF7, and MDA-MB-231 cells were seeded into 6-well plates and subjected to lentivirus infection (viral solution: medium = 1:1). In the infection process cells were incubated in polybrene (2 μg/Ml) in the incubator for a duration of 12 hours. Then the cultivation medium was replaced with a fresh one. After 72 hours of infection, the fusion rate of cells infected by virus infection was up to 80-90% under observation by fluorescence microscope. The cells were then passaged into multi-well plates for further culture. MCF7 and MDA-MB-231 cells were extracted for Western Blot to detect the infection efficiency.

### 2.13 RNA extraction and real-time PCR

TRIzol™ (TermoFisher, USA) was utilized to extract total RNA from the cells, and the front-strand cDNA synthesis kit (TaKaRa, Japan) was employed for reverse transcription, followed by real-time polymerase chain reaction (RT-PCR). The specific primers for KLRB1 and β-Actin were: KLRB1 forward primer 5’- GTTCCACCAAAGAATCCAGCCTG-3’ and reverse primer 5’- AAGAGCCGTTTATCCACTTCCAG-3’, β-Actin forward primer 5’- CACCATTGGCAATGAGGGTTCTC -3’ and the reverse primer 5’- AGGTCTTTGCGTGTCCACGT-3’.

### 2.14 MTT assay

The mixture of MCF7 and MDA-MB-231 cells were grown in 96-well plates at a density of 5000 cells in each well. Furthermore, the edge wells were filled with 200 µl sterile PBS. 96-well plates were kept in an incubator with different time gradients according to the experimental needs. Afterward, 20 μl of MTT solution (5 mg/ml, Beijing Zhongguang Ltd.) was introduced into all wells, and the cells were subsequently incubated for an additional 4 hours. The MTT solution was gently aspirated from each well. Subsequently, 150 μl of DMSO was introduced into all wells, and the plate was shaken slowly for 10 minutes to completely dissolve the methane. The 96-well plate was placed in an enzyme calibrator to read the OD value of each well at 570 nm.

### 2.15 Wound healing assay

MCF7 and MDA-MB-231 cells were inoculated in 6-well plates and allowed to culture overnight. Once the cell density reached 90%, the cell surface was scratched with a 1000 μl pipette tip. Images of the scratches were captured using an inverted microscope (CKX31, Olympus, Japan). Then, cells were treated with serum-free medium after lysis and incubated for 24 hours, and photographs of the scratches were taken at the same location using a microscope.

### 2.16 Transwell assay

Pre-chilled DMEM medium and matrix gel were drawn through the pipette tip to configure the matrix gel working solution (matrix gel: DMEM medium = 1:5). To begin, 50 µl of the matrix gel working solution was aspirated from the bottom of the well. The Transwell and 24-well plates were then incubated in an incubator for 2 hours, and the matrix gel was observed to confirm its solidification. Next, the cell concentration was regulated to 2.5 X 10^6^ cells/mL based on cell counting. Following this, 200 µL of cell suspension was aspirated into the Transwell, and 500 µL of 20% complete medium was introduced to the well plate to ensure that the liquid level in the wells was in contact with the liquid level in the well plate. The migration assay was carried through for 24 hours, and the invasion assay for 36 hours. Transwell plates were removed and washed thrice for 5 minutes with PBS. Following this, cells were fixed using 4% paraformaldehyde for 20 minutes, and then the washing step was repeated again. The cells were left to air dry and then stained with crystal violet for 30 minutes. Following staining, the washing step was repeated again, and the plates were inverted to air dry.

### 2.17 Cell cycle

The cells were cultured at a density of 10×10^4^ cells per well. After digestion with EDTA-free trypsin, the cells were collected by centrifugation, and the resulting supernatant was discarded. The cell suspension was gently agitated by adding pre-cooled PBS, followed by a second centrifugation step at 1800 rpm for 5 minutes. Afterward, the PBS was removed, and the cells were fixed overnight in 70% ice ethanol. The sample was centrifuged at 1800 rpm for 5 minutes, and the clear supernatant was discarded. Pre-cooled PBS was added to aspirate the suspended cells and the centrifugation step was executed again with subsequent discarding of the PBS. 100 µL of Rnase solution was utilized for resuspension of the cells, after which they were incubated at 37°C for 30 minutes. PI dye was introduced, and the cells were incubated for 30 minutes at 4°C before detection was performed.

### 2.18 Western blot

To prepare whole cell or tissue lysates, pre-cooled NP-40 buffer mixed with protease and phosphatase inhibitors (Roche, USA) was used, and the mixture was centrifuged at 12,000 × rpm at 4°C for 10 minutes. The resulting protein lysates were separated by SDS-PAGE and shifted to polyvinylidene fluoride (PVDF) membranes (Merck, Germany) which were incubated in 1× TBST buffer containing 5% non-skimmed milk powder for 2 hours at room temperature. PVDF membranes were incubated with the indicated primary antibodies and then washed with 1× TBST. Signal detection utilizing a Western ECL substrate kit (Bio-Rad, CA) was then performed using HRP-coupled secondary antibodies. The following antibodies were used for immunoblotting: KLRB1 (67537-1-Ig) and β-actin (81115-1-RR) were purchased from proteintech (China).

### 2.19 EdU Assay

Logarithmic growth phase cells were digested and centrifuged, each group of cells was adjusted to 3×104/mL with basal medium, 100ul was added to the plate with 5 side wells per group, and the cell adhesion was observed overnight. 100ul of EdU working solution per well and incubate in a cell culture incubator for 2 h. PBS was cleaned once, and 4% paraformaldehyde was added to fix at room temperature for 30min. Perforation of PBS solution of 0.3% TritonX and leave at room temperature for 15 min. Prepare the Click reaction solution according to the kit and add it to the well plate and incubate for 30 min at room temperature protected from light. Configure 1X Hoechst solution and incubate for 10 min at room temperature in the dark for nuclear staining. PBS is washed 3 times and post-photographed statistics are performed under a fluorescence microscope.

### 2.20 Statistical analysis

All experiments that required statistical analysis were conducted in triplicates. The experimental data were presented as mean ± standard deviation (mean ± SD). GraphPad Prism 9.0. was employed to execute the statistical analyses. Comparative assessment of two and multiple samples was executed through an unpaired Student’s t-test and a one-way analysis of variance (ANOVA), respectively, for variability. A *p*-value< 0.05 was considered a statistically significant value.

## 3 Results

### 3.1 Identifying DEGs and enrichment analysis of BC TME in accordance with stromal and immune scores

**Figure 1:**
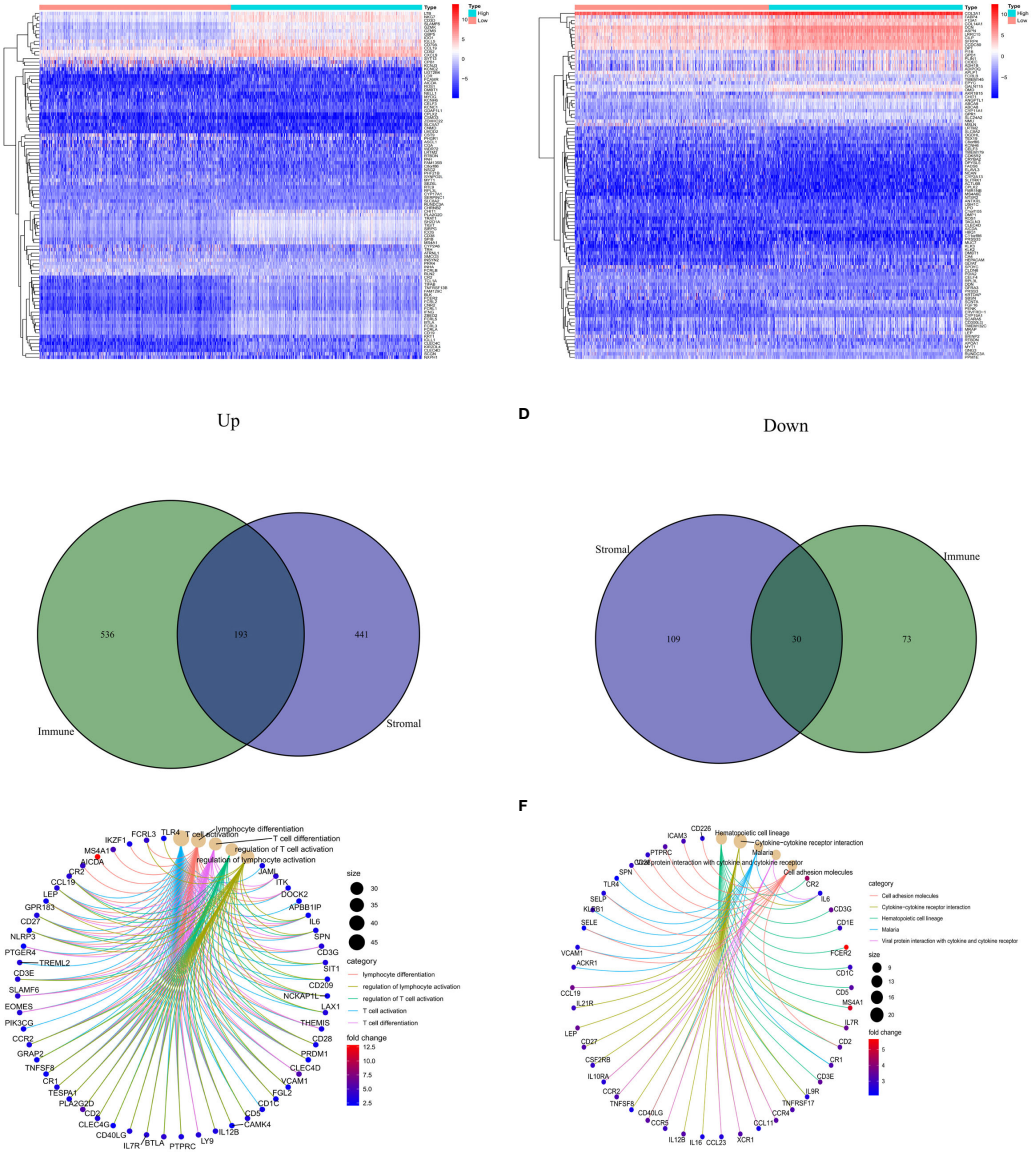
Comparison of stromal and immune scores in BC patients. **(A, B)** Heatmaps show the top 50 increases and downwards in immune scores and stromal scores. DEGs are tested using Wilcoxon rank and testing (P<0.05 |log2FC|<1). **(C, D)** Venn chart analysis of DEGs based on immune scores and stromalscores. **(E, F)** Analysis of biological functions and pathways associated with degs in BC, pValue <0.05 was considered significantly. BC: Breast cancer; DEGs: Different express genes.

Through the analysis of high- and low-scoring samples, it was found that DEGs of immune and stromal components have important roles in BC TME. The analysis identified a total of 832 DEGs through immune scoring, wherein 729 genes were overexpressed, and 103 genes had decreased expression (**Figure 1A**). In terms of stromal scoring, 773 DEGs were identified in total, comprising 634 upregulated genes and 139 downregulated genes (**Figure 1B**). A Venn diagram was used to identify DEGs associated with interstitial and prevalence scores, with 193 upregulated and 30 downregulated genes (**Figures 1C, D**). The identified DEGs could potentially be significant factors influencing the status of the TME. GO enrichment analysis highlighted that these DEGs are primarily involved in physiological processes such as T cell activation, differentiation, and regulation, as well as lymphocyte differentiation and regulation (**Figure 1E**). KEGG enrichment analysis also revealed that these DEGs help in cell adhesion molecule interactions, cytokine-receptor interactions, hematopoietic cell lineage, and viral proteins with cytokine and cytokine receptor interactions (**Figure 1F**).

### 3.2 Constructing and validating the stability of the prognostic model

**Figure 2:**
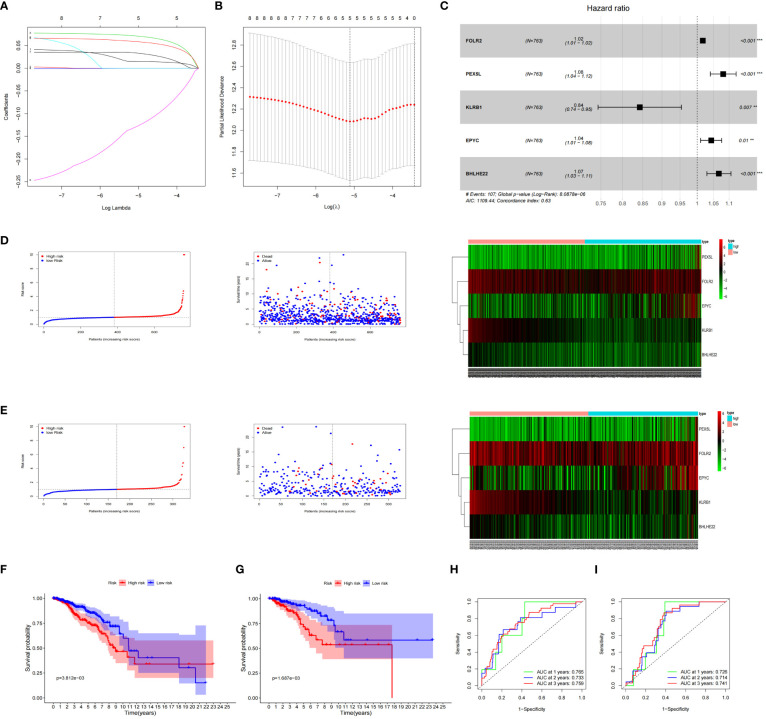
Prognosis of 5 genetic characteristic models in training and testing cohort. **(A)** LASSO regression of 5 os-related genes. **(B)** Cross-validated the method of adjusting parameter selection in LASSO regression. **(C)** Five gene forests map. Training cohort **(D)** and testing cohort **(E)** include median of the risk score, the status of OS and the expression spectrum of five immune genes. **(F, G)** Kaplan-Meier analysis survival rates for patients in high-risk and low-risk groups. **(H, I)** AUC time-dependent ROC curve evaluates the prognosis model for OS.

**Figure 3:**
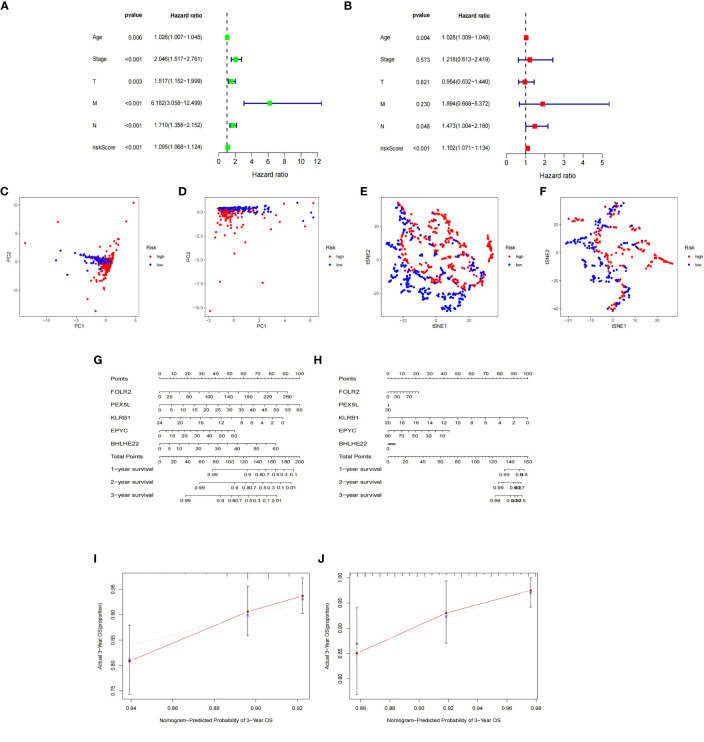
Nomogram based on the prognosis characteristics of the five genes is in the TCGA cohort. Univariate Cox **(A)** and multivariate Cox **(B)** regression analysis identified riskscore as a risk factor for breast cancer prognosis. PCA diagram **(C, D)** and t-SNE analysis **(E, F)** in Training cohort and testing cohort. Build a nomogram model of five genes and high and low risk to predict one, two, and three years of survival with Training cohort **(G)** and testing cohort **(H)**. The calibration chart shows that the predicted survival rate is consistent with the actual survival rates for 3 years with Training cohort **(I)** and testing cohort **(J)**.

The expression data of the 223 DEGs obtained were grouped according to the training set vs. validation set as 7:3. One-way Cox analysis was utilized to find nine prognosis-related DEGs. Subsequently, Lasso regression analysis was conducted on these nine DEGs to obtain the coefficients of five prognostic genes. (**Figures 2A, B**). Five prognostic genes (FOLR2, PEX5L, KLRB1, EPYC, and BHLHE22) that were significantly different in the multifactorial Cox regression model were identified (**Figure 2C**). For each BC patient, a risk score was quantified, and the individuals were classified per the median value into high-risk and low-risk groups. More individuals died in the high-risk group than those in the low-risk group. Conversely, the expression of KLRB1 was found to be elevated in the individuals in the low-risk group in comparison with the people in the high-risk group. (**Figures 2D, E**). According to Kaplan-Meier analysis, both the training and validation sets had a considerably increased overall survival rate for individuals belonging to the low-risk group in comparison with the individuals in the high-risk group (*P*<0.05) (**Figures 2F, G**). The respective three-year ROC curve areas for the training and validation sets were 0.759 and 0.741 (**Figures 2H, I**). The riskscore is an independent prognostic factor predicting breast cancer prognosis (**Figures 3A, B**). The results of both PCA analysis and t-SNE analysis indicated that individuals belonging to both risk groups were sorted into two distinct directions (**Figures 3C–F**). Multifactorial analyses were extracted from the training set and validation set data to create diagnostic curves to validate the scores of the five independent prognostic factors, the corresponding probabilities were found, and the survival probability of individuals at 1-, 2-, and 3- years was estimated (**Figures 3G, H**). The calibration plots demonstrated favorable performance in predicting the probability of survival beyond 3 years (**Figures 3I, J**).

### 3.3 Low expression of KLRB1 is correlated with poor prognosis in BC

**Figure 4:**
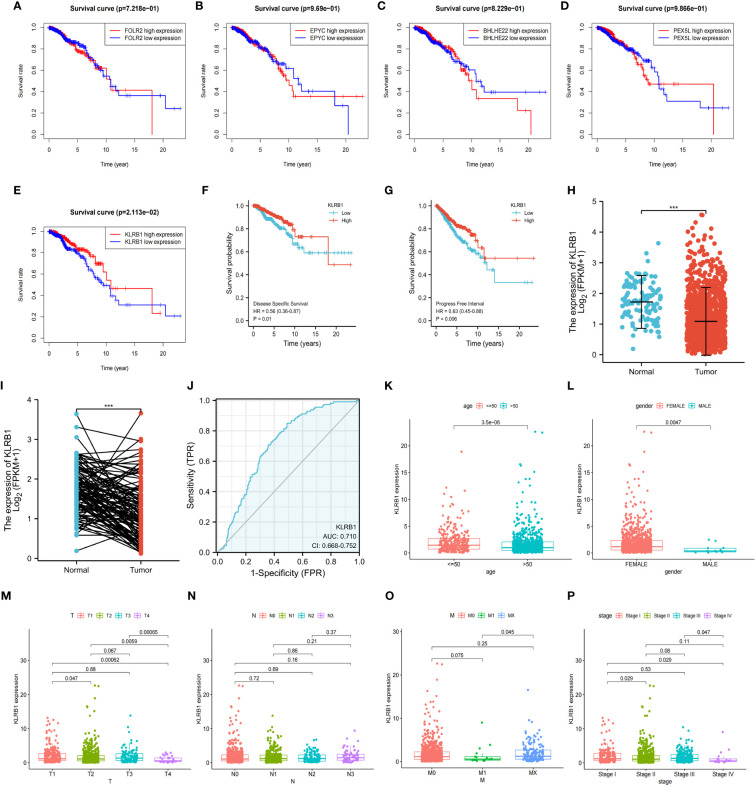
The relationship between the expression and total survival of 5 genes in BC. **(A–E)** The expression of five genes is associated with the overall survival prognosis of breast cancer. **(F, G)** The K-M analysis of KLRB1 with DSS and PFI in breast cancer patients. **(H)** KLRB1 mRNA is expressed at a low level in BC tissues. **(I)** Evaluate the level of KLRB1 mRNA in paired BC tissues. **(J)** Construct a ROC curve to predict the impact of KLRB1 on the overall survival of breast cancer patients. **(K–P)** Investigate the expression of KLRB1 in breast cancer patients with respect to age, gender, T size, N stage, M stage, and Stage. DSS, Disease Special Survival. PFI,Progression Free Interval.

To investigate the overall survival (OS) of the five genes related to prognosis, a Kaplan-Meier analysis was conducted, which revealed that individuals having reduced expression of KLRB1 had a remarkably shorter OS. However, the expression of the remaining four genes did not show any association with OS (**Figures 4A–E**). Then we found that KLRB1 was strongly associated with DSS and PFI in breast cancer (**Figures 4F, G**). The expression of KLRB1 was considerably lower in BC tissues (**Figures 4H, I**). The area under the ROC curve (AUC) for KLRB1 as a predictor of OS in BC was 0.71 (**Figure 4J**). The expression of KLRB1 depicted a positive association with patient age, gender, tumor size, and tumor stage (**Figures 4K–P**). We also assessed baseline data on high and low expression of KLRB1 in breast cancer. The KLRB1 is closely related to age, clinical stage, pathological type, estrogen receptor and molecular typing of breast cancer (**Table 1**). Considering that KLRB1 expression correlates with the molecular typing of breast cancer, we further analyzed and found that KLRB1 was also associated with the prognosis of Luminal A and Luminal B subtypes, but not statistically significant with HER-2 positivity and TNBC survival (**Figures S1A–D**). Finally, we also analyzed the correlation analysis of KLRB1 with ESR1, PGR, and ERBB2, and found that there was a negative correlation with ESR1, PGR and ERBB2 (**Figures S1E–G**).

**Table 1:** Baseline data sheet about KLRB1 express.

Characteristic	Low expression of KLRB1	High expression of KLRB1	p
n	541	542	
Age, n (%)			< 0.001
<=60	268 (24.7%)	333 (30.7%)	
>60	273 (25.2%)	209 (19.3%)	
T stage, n (%)			0.142
T1	132 (12.2%)	145 (13.4%)	
T2	323 (29.9%)	306 (28.3%)	
T3	61 (5.6%)	78 (7.2%)	
T4	22 (2%)	13 (1.2%)	
N stage, n (%)			0.182
N0	270 (25.4%)	244 (22.9%)	
N1	172 (16.2%)	186 (17.5%)	
N2	52 (4.9%)	64 (6%)	
N3	32 (3%)	44 (4.1%)	
M stage, n (%)			0.044
M0	448 (48.6%)	454 (49.2%)	
M1	15 (1.6%)	5 (0.5%)	
Pathologic stage, n (%)			0.025
Stage I	86 (8.1%)	95 (9%)	
Stage II	323 (30.5%)	296 (27.9%)	
Stage III	105 (9.9%)	137 (12.9%)	
Stage IV	13 (1.2%)	5 (0.5%)	
Histological type, n (%)			< 0.001
Infiltrating Ductal Carcinoma	416 (42.6%)	356 (36.4%)	
Infiltrating Lobular Carcinoma	66 (6.8%)	139 (14.2%)	
ER status, n (%)			0.021
Negative	102 (9.9%)	138 (13.3%)	
Indeterminate	1 (0.1%)	1 (0.1%)	
Positive	410 (39.6%)	383 (37%)	
PR status, n (%)			0.244
Negative	159 (15.4%)	183 (17.7%)	
Indeterminate	1 (0.1%)	3 (0.3%)	
Positive	352 (34%)	336 (32.5%)	
HER2 status, n (%)			0.236
Negative	256 (35.2%)	302 (41.5%)	
Indeterminate	6 (0.8%)	6 (0.8%)	
Positive	84 (11.6%)	73 (10%)	
PAM50, n (%)			< 0.001
Normal	7 (0.6%)	33 (3%)	
LumA	277 (25.6%)	285 (26.3%)	
LumB	134 (12.4%)	70 (6.5%)	
Her2	36 (3.3%)	46 (4.2%)	
Basal	87 (8%)	108 (10%)	

### 3.4 Validation and enrichment analysis of the KLRB1 model in BC

**Figure 5:**
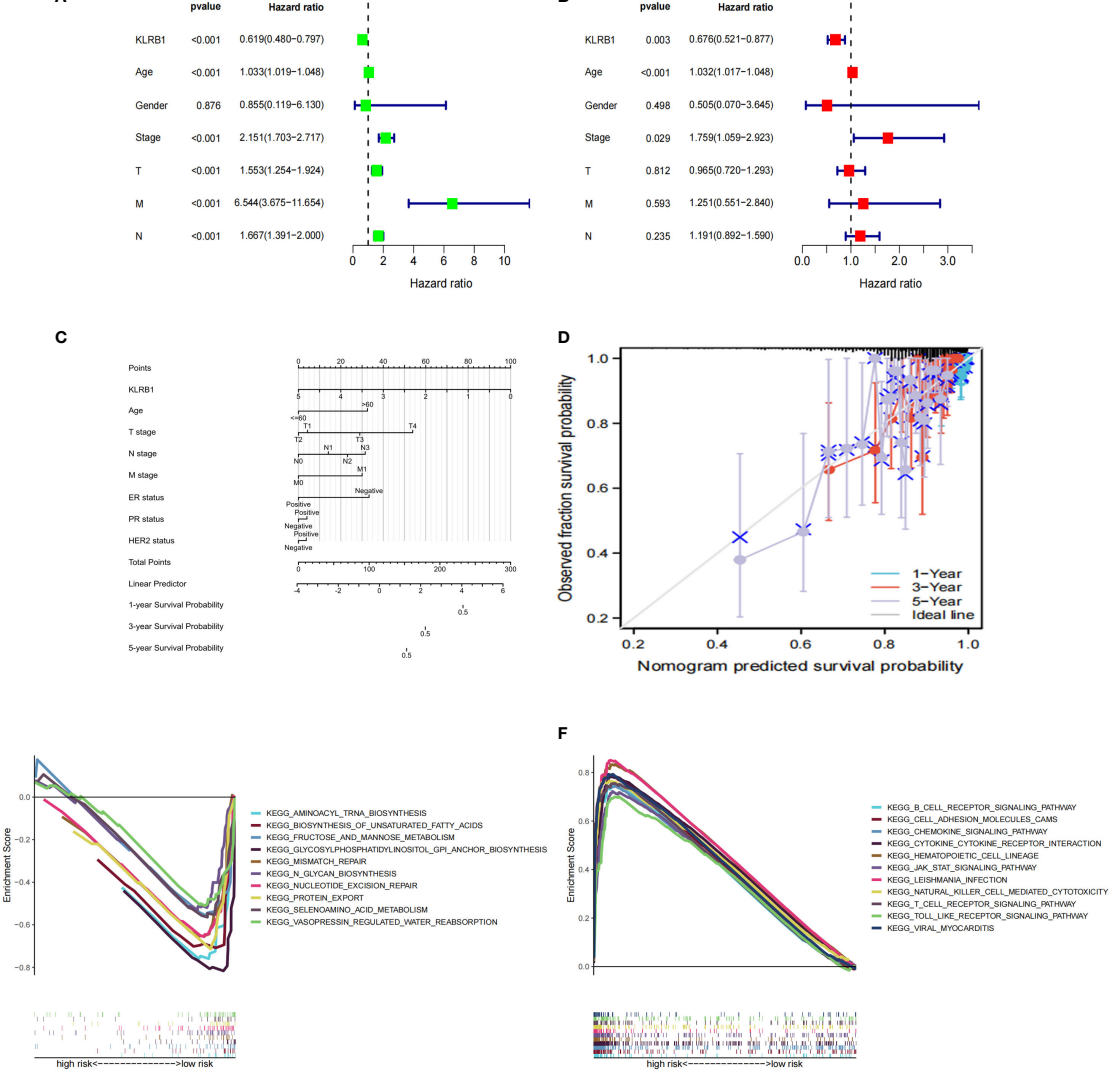
The prognostic value of KLRB1 in breast cancer. **(A, B)** Univariate and multivariate Cox regression analysis. **(C)** Nomogram predicting the probability of 1-year, 3-year, and 5-year OS for risk score and clinical characteristics. **(D)** Calibration curves of the nomogram for predicting of 1-, 3-, and 5-year OS in all BC patients. GO and KEGG enrichment analysis of KLRB1 associated genes. OS, overall survival. **(E)** The genome showing high expression of KLRB1 exhibited notable enrichment in immune-related activities. **(F)** The KLRB1 low expression genome was mainly enriched in metabolic and biosynthetic pathways.

Both univariate and multivariate Cox regression analyses highlighted that KLRB1 served as an independent prognostic factor in BC (**Figures 5A, B**). Based on KLRB1 and clinicopathological features, a prognostic scale was developed for the prediction of the prognosis of BC (**Figure 5C**). The calibration curve was approximately diagonal, indicating that the prognostic scoring scale had strong predictive power for survival at 1-, 3-, and 5- years (**Figure 5D**). The genome showing high expression of KLRB1 exhibited notable enrichment in immune-related activities, such as cell adhesion and signaling pathways including chemokine, T cell receptor, B cell receptor, NK cell regulatory, and JAK/STAT signaling pathway (**Figure 5E**). The KLRB1 low expression genome was mainly enriched in metabolic and biosynthetic pathways, including unsaturated fatty acid biosynthesis, phosphatidyl inositol acyl-anchored biosynthesis, N-glycosylated biosynthesis, fructose and mannitol metabolism, and selenium amino acid metabolism (**Figure 5F**). In conclusion, these outcomes suggested that KLRB1 might be a potential indicator in TME.

### 3.5 KLRB1 affects the expression of immune cells in BC TME

**Figure 6:**
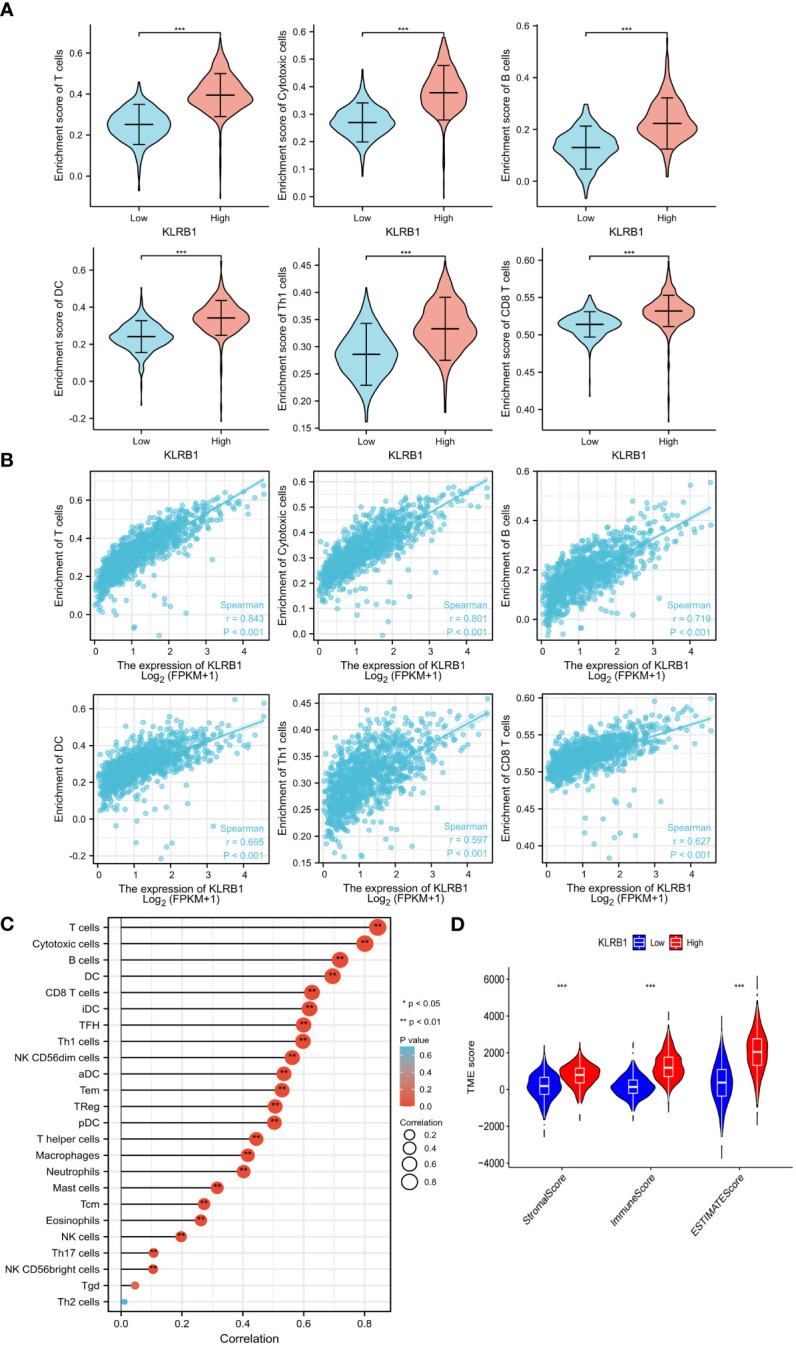
KLRB1 affects immune cell expression in the TME in breast cancer. The relationship between KLRB1 expression and the enrichment scores of different immune-infiltrating cells **(A)**. The abundance of immune-infiltrating cells **(B)**. The correlation of KLRB1 with 22 immune cells in the breast cancer tumor microenvironment (TME) are investigated **(C)**. The association between KLRB1 expression and immune scores, stromal scores, and ESTIMATE scores is examined **(D)**. *P<0.05, **P<0.01, ***P<0.001.

To investigate the potential role of KLRB1 in the tumor microenvironment, a cell sorting algorithm was employed to detect the proportion of 22 immune cells present in the BC microenvironment (**Figure 6C**). In addition, the group with high KLRB1 expression was scored for immunity using the ESTIMATE procedure, having a considerably higher immunity score, stromal score, and ESTIMATE score (**Figure 6D**). In addition, the Wilcoxon-Mann Whitney test showed that T cells, B cells, CD8 T cells, Th1 cells, DC cells, and cytotoxic cells were relatively high in the high KLRB1 expression group and relatively low in the low KLRB1 expression group (**Figure 6A**). Expression of KLRB1 was linked positively with the abundance of innate immune cells (**Figure 6B**), including T cells (r = 0.843), cytotoxic cells (r = 0.801), B cells (r = 0.719), Th1 cells (r = 0.597) and DC (r = 0.695), and CD8 T cells (r = 0627) (all *P*< 0.001).

### 3.6 KLRB1 expression associated with chemotherapy sensitivity and immunotherapy response

Specifically, patients with low expression of KLRB1 were found to be more sensitive to doxorubicin, paclitaxel, docetaxel, and 5-fluorouracil (**Figures S2A–D**). Currently, immunotherapy for breast cancer mainly focuses on PD-L1 and CTLA4. Interestingly, our study revealed that when both CTLA4 and PD-L1 were positive or when either CTLA4 or PD-L1 was positive, patients with low KLRB1 expression exhibited lower expression levels of both CTLA4 and PD-L1 than those who were negative for both CTLA4 and PD-L1 (**Figures S2E–H**).

### 3.7 Validation of KLRB1 *in vitro* assays

**Figure 7:**
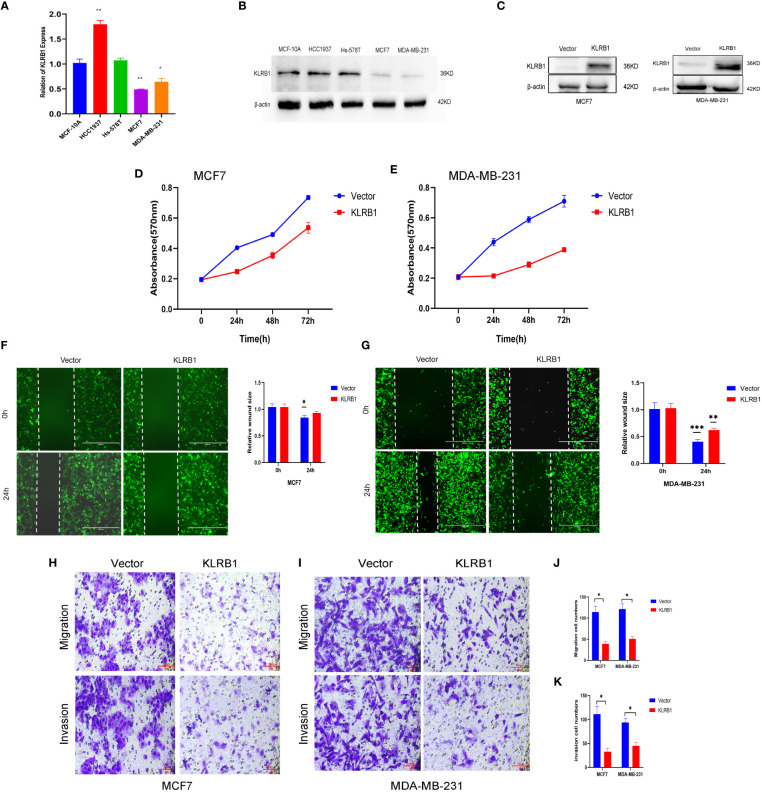
KLRB1 is involved in cell proliferation and migration in breast cancer cells. **(A)** qPCR confirmed KLRB1 was low expression in MCF7 and MDA-MB-231 cells. **(B)** KLRB1 were low express in MCF7 and MDA-MB-231 cells. **(C)** Western Blot verifies KLRB1 overexpression. **(D, E)** Overexpression of KLRB1 inhibits the proliferative activity of MCF7 and MDA-MB-231 cells. **(F, G)** KLRB1 inhibits the migration ability of MCF7 and MDA-MB-231 cells. **(H-K)** KLRB1 inhibits the migration and invasion ability of both MCF7 and MDA-MB-231 cells. *P<0.05, **P<0.01, ***P<0.001.

**Figure 8:**
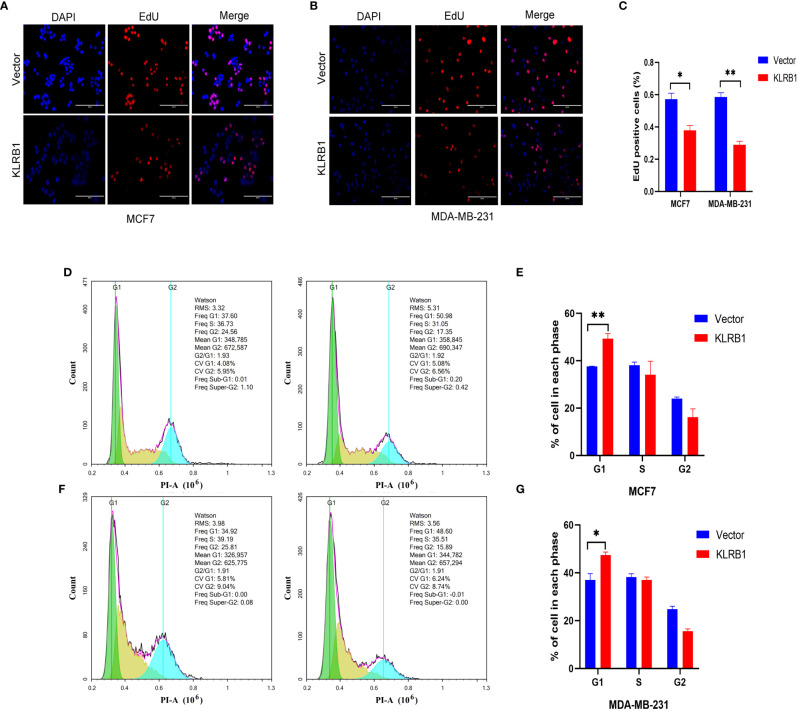
KLRB1 affects DNA replication and changes cell cycle in breast cancer cells. **(A–C)** KLRB1 inhibits the DNA replication ability of MCF7 and MDA-MB-231 cells. **(D, E)** KLRB1 blocks MCF7 cells in the G1 phase. **(F, G)** KLRB1 blocks MDA-MB-231 cells in the G1 phase. *P<0.05, **P<0.01.

The qPCR experiments confirmed that KLRB1 expression was low in MCF7 and MDA-MB-231 cells (**Figure 7A**). Then we demonstrated that the KLRB1 protein is significantly lower than normal breast epithelial cells in breast cancer MCF7 and MDA-MB-231 cells (**Figure 7B**). After infection with KLRB1 lentivirus, we verified KLRB1 overexpression in MCF7 and MDA-MB-231 cells, confirming that BC cell lines stably expressing KLRB1 were constructed (**Figure 7C**). The MTT assay results demonstrated that the overexpressed KLRB1 significantly suppressed the proliferation and viability of both MCF7 and MDA-MB-231 cells (**Figures 7D, E**). The scratch assay results showed that KLRB1 could considerably inhibit the migrative capacity of MCF7 and MDA-MB-231 cells (**Figures 7F, G**). While the inhibition of their invasive and migrative abilities by KLRB1 was determined through Transwell assay (**Figures 7H–K**). Meanwhile, the EdU assay showed that KLRB1 inhibits the DNA replication capacity of cells (**Figures 8A–C**). Flow cytometry assays showed that KLRB1 can block MCF7 cells (**Figures 8D, E**) and MDA-MB-231 cells in the G1 phase (**Figures 8F, G**). The data suggested that KLRB1 might inhibit the proliferation of BC cells by preventing cells from dividing in the G1 cycle.

## 4 Discussion

The objective of this research was to identify TME genes from the TCGA-BC database that could be used for the diagnosis and staging of TNM and prediction of OS in BC patients. The close association between KLRB1 and immune activity in TME was verified, highlighting its potential as a therapeutic target for BC patients. TMEs formed by tumors are dynamically unstable environments regulated by tumors. Their composition and function change at different stages of tumor development and are closely associated with tumor prognosis and overall disease process (21). The TME provides a favorable growth environment for tumors and promotes their malignant proliferation. However, they also interfere with the function of immune and stromal cells in the microenvironment, leading to tumor evasion of immune surveillance, promotion of metastasis, and drug tolerance (22). These findings highlight the significance of studying the interactions between tumor cells and immune cells and provide new perspectives for the development of more efficient therapeutic options. This analysis identified five immune-related genes, namely FOLR2, PEX5L, KLRB1, EPYC, and BHLHE22, that have prognostic significance.

Belonging to the soluble folate receptor family, FOLR2 is primarily expressed in the placenta, hematopoietic cells, and macrophages, where it is anchored to the extracellular surface by GPI (23). FOLR2 exhibits high expression levels in tumor-associated macrophages (TAMs) of ovarian cancer and can be selectively depleted by G5-MTXNps. In a mouse model of ovarian cancer, TAM depletion inhibited tumor growth. Furthermore, TAM depletion is linked with angiogenesis, which can overcome resistance to VEGF-A therapy when G5-MTXNps is combined with anti-VEGF-A therapy. Therefore, targeting FOLR2 in TAM could be a potential treatment for cancer patients (24). PEX5L is correlated with LINC00924 and serves as an independent predictor of peritoneal metastasis in gastric cancer. This finding indicates that targeting LINC00924/PEX5L could be a potential strategy for molecular targeted therapy (25). EPYC exhibits high expression in ovarian cancer and is significantly associated with both OS and disease-free survival (DFS) in patients with ovarian cancer (26). BHLHE2 methylation is increased considerably in healthy endometrium, endometrial hyperplasia, and type I and type II endometrial cancer, and might be a potential molecular target for predicting cervical cancer (27). In a comprehensive analysis of the entire cancer genome, the gene KLRB1 encoding CD161 showed a good clinical prognosis in breast, colorectal, prostate, melanoma, and neuroblastoma carcinomas (28–32).

The development of BC involves genetic and epigenetic changes in multiple genes. The analysis of multi-genomics (transcriptome, microbiome, epigenome, metabolome, and proteome) at different cellular levels provides new perspectives on the formation, diagnosis, and prognosis of BC. With the development of high-throughput technologies, the various mutations, methylation, copy number, and gene expression patterns have been identified for various cancer types. Copy number variation (CNV) is frequently regarded as a form of genetic variation and is involved in the pathogenesis of BC (33). BRCA1 and BRCA2 are the major BC-related genes, and women carrying BRCA1/2 mutations have a significantly increased risk of BC (34). DNA methylation is a crucial epigenetic modification that regulates gene transcription and maintains genomic stability. Altered methylation, commonly characterized by hypermethylation of proto-oncogenes and methylation of tumor suppressor genes, is critically involved in regulating gene expression in BC (35). The findings of this study suggested that aberrant KLRB1 expression might be due to a combination of copy number variants and methylation variants. Moreover, multi-omics analysis of these genes can help us better understand the molecular mechanisms linked with the development and progression of BC.

The study revealed that only KLRB1 was linked to prognosis in BC patients and had the potential to serve as a biomarker for BC. Individuals with elevated KLRB1 expression had longer survival rates compared to those with low KLRB1 expression. In stage T4 tumors, KLRB1 expression was significantly decreased, suggesting that decreased KLRB1 expression leads to the possibility of poor prognosis in patients, which is consistent with the findings of survival analysis. The above results suggest that KLRB1 expression is closely linked with clinicopathological parameters and poor prognosis. It implied the possible function of KLRB1 as a prognostic marker and therapeutic target for TME in BC. Hence, further analysis was executed to assess the link between KLRB1 expression and TME. The outcomes of GSEA highlighted that the group with over-expression of KLRB1 was mainly concentrated in the cell adhesion (36), and signaling pathways such as B cell receptor, T cell receptor (37), chemokine (38), JAK-STAT, VEGF signaling pathways, and other tumor development-associated pathways.

In this study, CIBERSORT analysis of TIC ratios in BC patients showed a positive correlation between T cells, cytotoxic cells, B cells, Th1 cells, DC cells, and CD8 T cells. The above immune effector cells are mainly responsible for cancer immunosurveillance. CD8 T cells are the primary effectors of the antitumor immune response with potent antitumor activity, and BC patients with high CD8 T cell expression generally have a more favorable prognosis (39), which is in agreement with the outcomes of the previous survival analysis. The analysis of tumor-associated macrophages, a primary component of the tumor stroma, and M2-type TAM concentrations within hypoxic tumor regions were conducted as they exhibit pro-angiogenic activity, and their levels increase with tumor progression (40). These findings align with the GSEA enrichment outcomes and provide further evidence of the validity of the conclusions of this study.

Based on these results, a link between the number of tumor immune infiltrating cells and BC survival can be demonstrated. This means maybe we can improve the prognosis of BC patients by targeting KLRB1 to eliminate the suppression of the immune microenvironment and enhance the immune response.

## 5 Conclusion

In conclusion, a series of bioinformatic analyses of BC samples from the TCGA database was performed, utilizing the ESTIMATE algorithm to determine genes associated with the TME. The association of KLRB1 with the tumor microenvironment of BC highlights that it can be utilized as a promising prognostic marker and therapeutic target. These findings offer a new direction for BC treatment.

## Data availability statement

The datasets presented in this study can be found in online repositories. The names of the repository/repositories and accession number(s) can be found below: https://www.jianguoyun.com/#/sandbox/16cf098/3fb9b64503eda5cf/%2/.

## Author contributions

JC, BJ and KX designed the project. GH, SX, SZ, ZJ, XZ and LC wrote the paper. GH, SX, ZJ, LL and KX perform bioinformatics analysis and MTT Assay, Wound healing Assay, Transwell Assay, Western Blot, qPCR, Cell Cycle Assay. JC, BJ and KX have rigorously revised the final manuscript. All authors also read and agree to release versions of the manuscript.
