# Supplementary figures and images for: Machine learning immune-related gene based on KLRB1 model for predicting the prognosis and immune cell infiltration of breast cancer
Source: Front Endocrinol (Lausanne). 2023 Jun 7;14:1185799. doi: 10.3389/fendo.2023.1185799 (PMC10282768; doi:10.3389/fendo.2023.1185799)

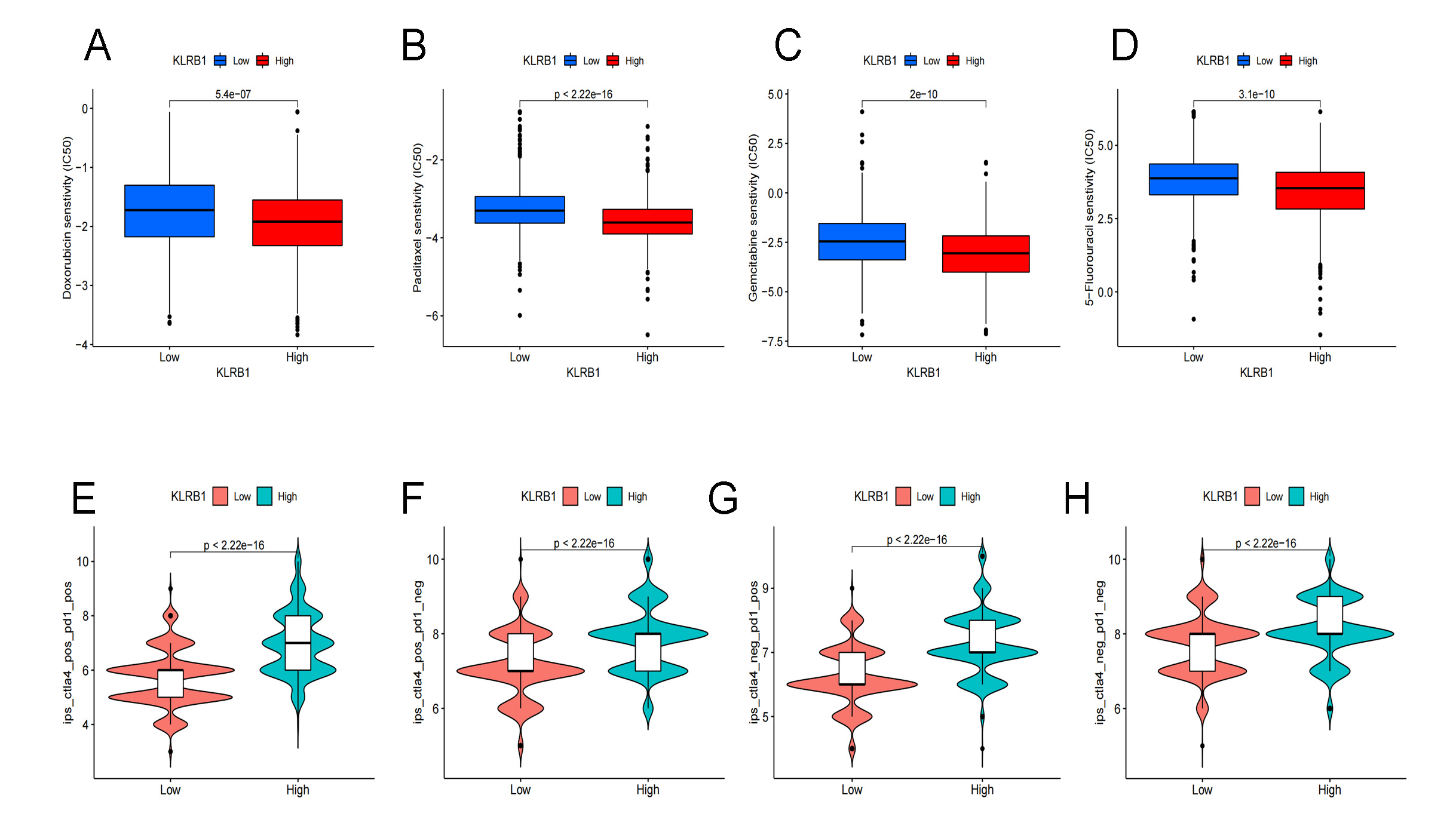

Supplement: Supplementary file 1 [file DataSheet_1.zip › Figure S2.jpg]

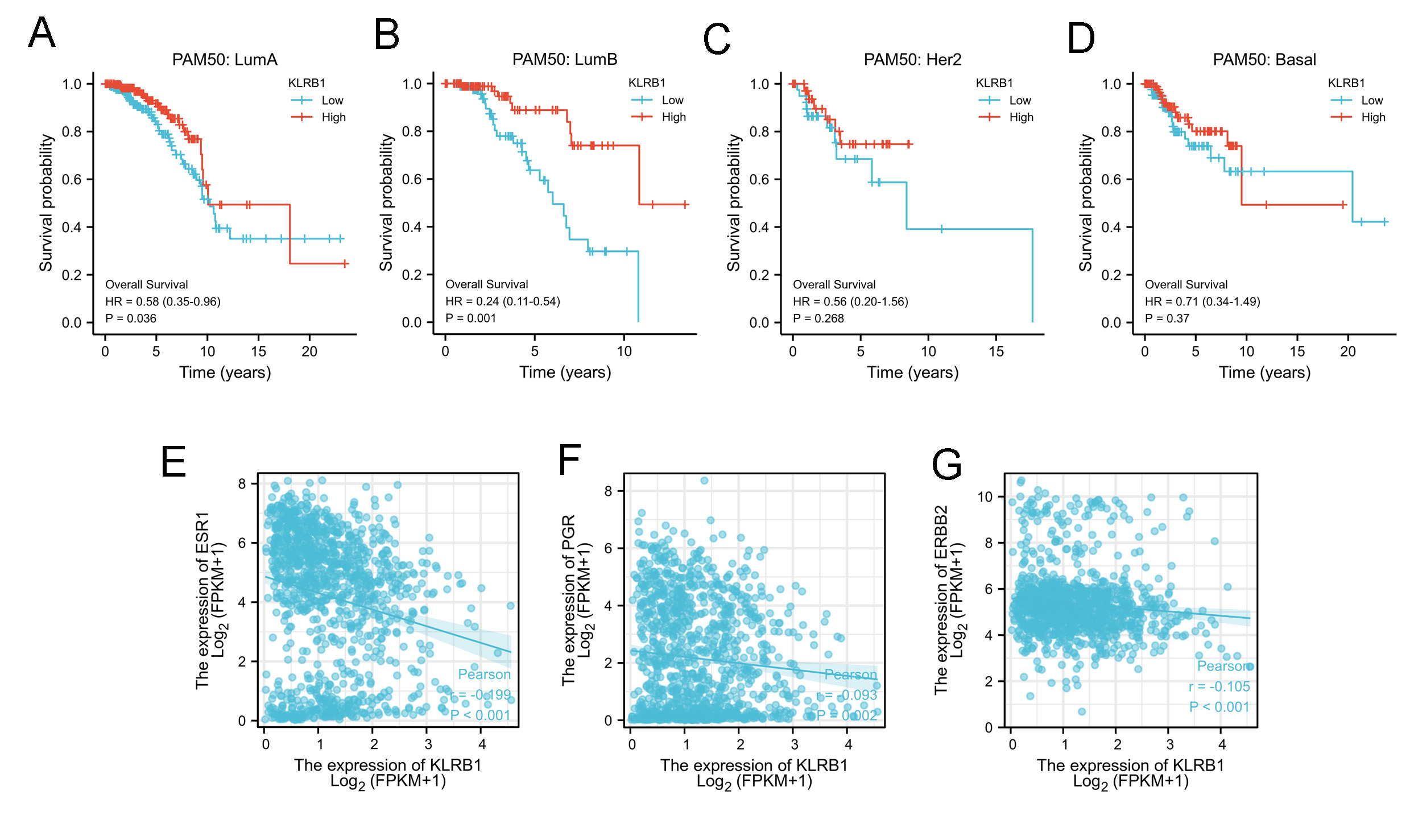

Supplement: Supplementary file 1 [file DataSheet_1.zip › Figure S1.jpg]
