# Supplementary material for: Machine learning immune-related gene based on KLRB1 model for predicting the prognosis and immune cell infiltration of breast cancer
Source: Front Endocrinol (Lausanne). 2023 Jun 7;14:1185799. doi: 10.3389/fendo.2023.1185799 (PMC10282768; doi:10.3389/fendo.2023.1185799)
Supplement: Supplementary file 1 [file DataSheet_1.zip › Figure supplementary .doc]

**Figure S1. Correlation between KLRB1 and breast cancer molecular typing and biomarkers.** （A）Luminal A, （B）Luminal B, （C）HER2, （D）Basal, （E-G）KLRB1 was negative correlation with ESR1, PGR and ERBB2.

**Figure S2**. **KLRB1 expression associated with chemotherapy sensitivity and immunotherapy response.** （A-D）KLRB1 low expression be more sensitive to doxorubicin, paclitaxel, docetaxel, and 5-fluorouracil. （E-H）Patients with positive PD-1 and CTLA4, positive CTLA4 or PD-1, and patients with negative PD-1 and CTLA4 have a higher sensitivity for immunotherapy with high expression of KLRB1.
